# White Light-Emitting Diodes Based on Individual Polymerized Carbon Nanodots

**DOI:** 10.1038/s41598-017-12083-2

**Published:** 2017-09-22

**Authors:** Zheng Xie, Zhengmao Yin, Yongzhong Wu, Chunyan Liu, Xiaopeng Hao, Qingqing Du, Xiangang Xu

**Affiliations:** 1 Laboratory of Photochemical Conversion and Optoelectronic Materials, Technical Institute of Physics and Chemistry, Chinese Academy of Sciences, No.29 Zhongguancun East Road, Haidian District Beijing, 100190 P. R. China; 2 State Key Laboratory of Crystal Materials, Shandong University, 27 Shandanan Road, Jinan, 250100 P. R. China; 3 College of Materials Science and Engineering, Qingdao University of Science and Technology, Qingdao, 266042 P. R. China; 4 Shandong Inspur HuaGuang Optoelectronics CO., LTD, 1835 Tianchen Street, High-tech Zone, Jinan, 250101 P. R. China

## Abstract

A search for new phosphor materials that exhibit high light-emission, spectral purity, long-time stability and processability capture particular attention to modern solid-state lighting. Here, polymerizable silane pre-functionalized carbon dot (SiCD) fluids were dripped and co-polymerized or completely bulk polymerized to build color conversion and encapsulation coatings of commercially available GaN blue LEDs. Most parameters of SiCD-based white LEDs were similar to or even better than those of phosphor-based white LEDs, particularly the insensitivity to excitation wavelength and working current. Thus, SiCDs were superior to those phosphors in terms of broadband properties, high transparency (no light blocking and leaking), as well as arbitrary doping of its content as color conversion and encapsulation layers simultaneously, unique solubility, flexible chemical, optical and mechanical processability. Thus, designing new CD-based white LEDs, instead of inorganic rare earth phosphor-based LEDs, is possible for better performance solid state lighting devices.

## Introduction

The development of white and full-color light-emitting technology is significant for display and lighting. Among them, light-emitting diodes (LEDs) have dominated lighting study and market share because of their energy efficiency, long-lifetime, reliability, and wide application range, such as in LED-based solid-state lighting^1^. In general, commercial white LEDs (WLEDs) are generated by combining a blue LED (2014 Nobel Prize in Physics) with a color conversion layer (CCL) such as yellow phosphors for dichromatic (yellow & blue) WLED, and recently, red and green phosphors for trichromatic (red, green & blue) WLED, to realize a high color rendering index (CRI) Ra and color tuning. However, today’s traditional phosphors, delivering up to 150–230 lm/W luminous efficiency LEDs^1^, rely solely on using combinations of rare-earth ions. The use of most of these rare-earth based phosphors is restricted by some intrinsic optical defect (relatively low color quality, light blocking and light leakage for microscale phosphors, and performance degradation for nanoscale phosphors), limited and hard preparation conditions (>1200 °C), high cost, toxicity and pollution in mining and refining, as well as requirement of international export and limited resource of rare-earth materials^2–5^.

Accordingly, a key and challenging assignments essential to modern solid-state lighting is searching for new CCL phosphor materials that exhibit high emission quantum yields (QY), spectral purity, long-term photo-stability, and long-term thermal-stability and good processability as well as breakup of monopoly to^6–11^. Since the same year (1996) of founding of blue LED and WLED, many efforts have been devoted to developing new CCLs, such as photoluminescent (PL) organic and polymers^2–5^, quantum dots (QDs)^6–24^, and carbon dots (CDs)^25–33^ for WLED applications. Organopolymer CCLs have already attracted significant interest thanks to their broad absorption & emission, moderate price, and ease of fabrication, but concerns over their stability. Recently, Bae *et al*. reported dye-bridged nanohybrid CCLs^5^, in which red and green-emitting silane-functionalized dyes were covalently linked to functional oligosiloxane. So, trichromatic nanohybrids exhibit broad color tunability and high CRI for white LED. However, four silane-functionalized dyes and organic compounds are also required by this system.

Since their inception 20 years ago, electrically driven QD-LEDs in which QD is used as an electroluminescence layer (ELL), have increased in external quantum efficiency from < 0.01% to 20.5%^7,8^. Consequently, many efforts have been focused on the same QD materials used as CCLs^3–6^. For the above-mentioned two applications, one challenge is the reduction of the toxicity of QDs considering that most successful examples are cadmium-based. Hence, developing other possible candidates is needed, such as InP, ZnTe QDs or VI QD (silicon QDs^21–24^, CDs^25–34^, etc.), most of them suffer from relatively low stability and moderate QY^11^. Another important challenge is the compatibility of QDs with encapsulation matrix typically used in the LED industry^10–24^. To solve this problem, which could lead to surface defects and aggregation, QDs must have an appropriate surface encapsulating modification and protecting shells so QDs will be dispersed in encapsulation silicone resin without any deterioration of stability, PL and other optical properties^13–17^. These steps can risk losing the high performance and doping concentration of QDs, which are critical factors in many applications^13,35^. In addition, all of these abovemetioned CCL materials are less green and environmental or stablity.

As the latest form of carbon nanomaterials and novel green QDs, CDs is a promising alternative to conventional organic dyes, QDs, and rare-earth materials in terms of chemical inertness, excellent photostability, simple synthesis, low cost, eco-friendliness, and very easy surface functionalization^35–39^. CDs can be prepared in a large amount with low cost and under moderate preparation conditions by decomposing of rich-carbon organics, natural matter, or abundant raw materials. Furthermore, the size and molecular weight of CDs is small and controllable. Some of them even show size-quantization effect in recently reports^40–45^. They can also be easily surface functionalized by various functional groups and imparted with excellent suitability and solubility for subsequent functionalization with various units. Thus, CDs can be used in a wide-ranging technologies, but tangible applications was not achieved expect for the potential bioimaging and sensor since their discovery in 2004^40^.

Among them, the performance of CDs is superior in terms of excellent luminescence such as remarkable photostability and chemical stability, high luminescent efficiency, as well as the broadly adjustable spectrum of absorption, excitation and emission. CDs can simultaneously act as donors and acceptors show a slow “thermal” carrier relaxation, and can easily form effective electron transferring, contributing to their optoelectronic conversation and photocatalysis. To date, a few studies have been performed and a proof-of-concept has demonstrated that CDs could achieve white^46,47^ and multicolor^40–45^ light emission and be used as an active material in optoelectronic devices, such as ELL of OLED^25–28^, and CCL of InGaN LED^28–34^. We have accomplished direct white-light emission based on CDs’ PL under laser excitation^46^ and WLED devices based on CDs’ EL, but very high operation voltage is required to obtain reasonable brightness^25^. Chen^28^ and Lau^32,33^ *et al*. reported CD-based LEDs showing limited absorption in deep-blue (260–300 nm) region. Thus, InGaN blue LEDs with wavelengths between 450 and 460 nm, which are the most commonly used and cost-effective base LEDs, cannot be effective. Furthermore, the performance of CD-based LEDs significantly limits because aggregation of solid-state CDs typically leads to serious PL quenching^31^. Therefore, a better understanding of CDs must be gained to guide the design and optimization of their use in lighting devices^34^.

**Figure 1:**
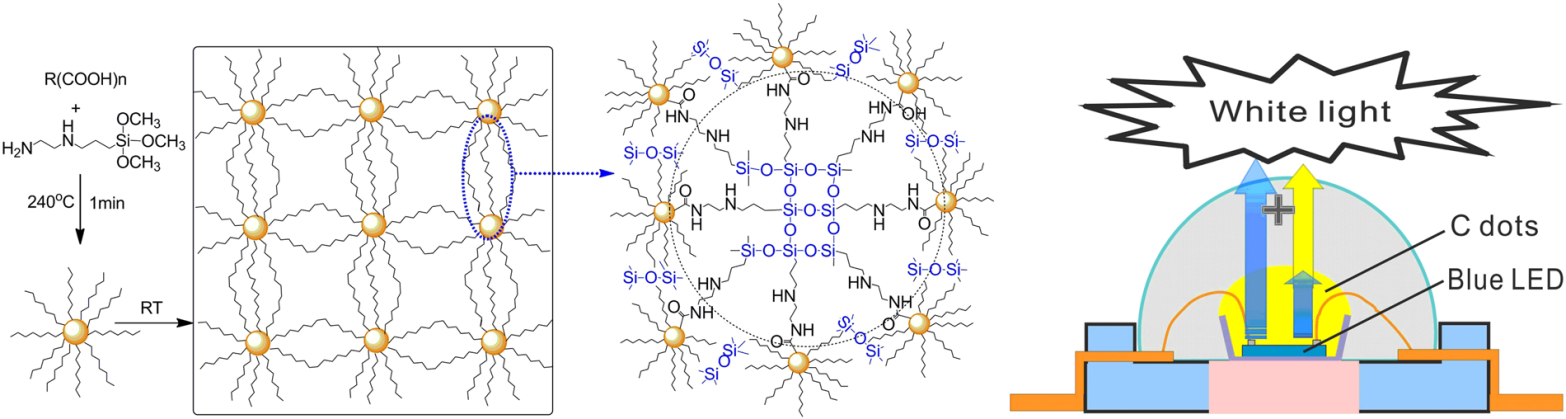
Structures of SiCD-based WLED. Schematic diagrams of chemical (left) and device structures (right) of self-polymerized silane-functionalized carbon dot layers on GaN LED.

**Figure 2:**
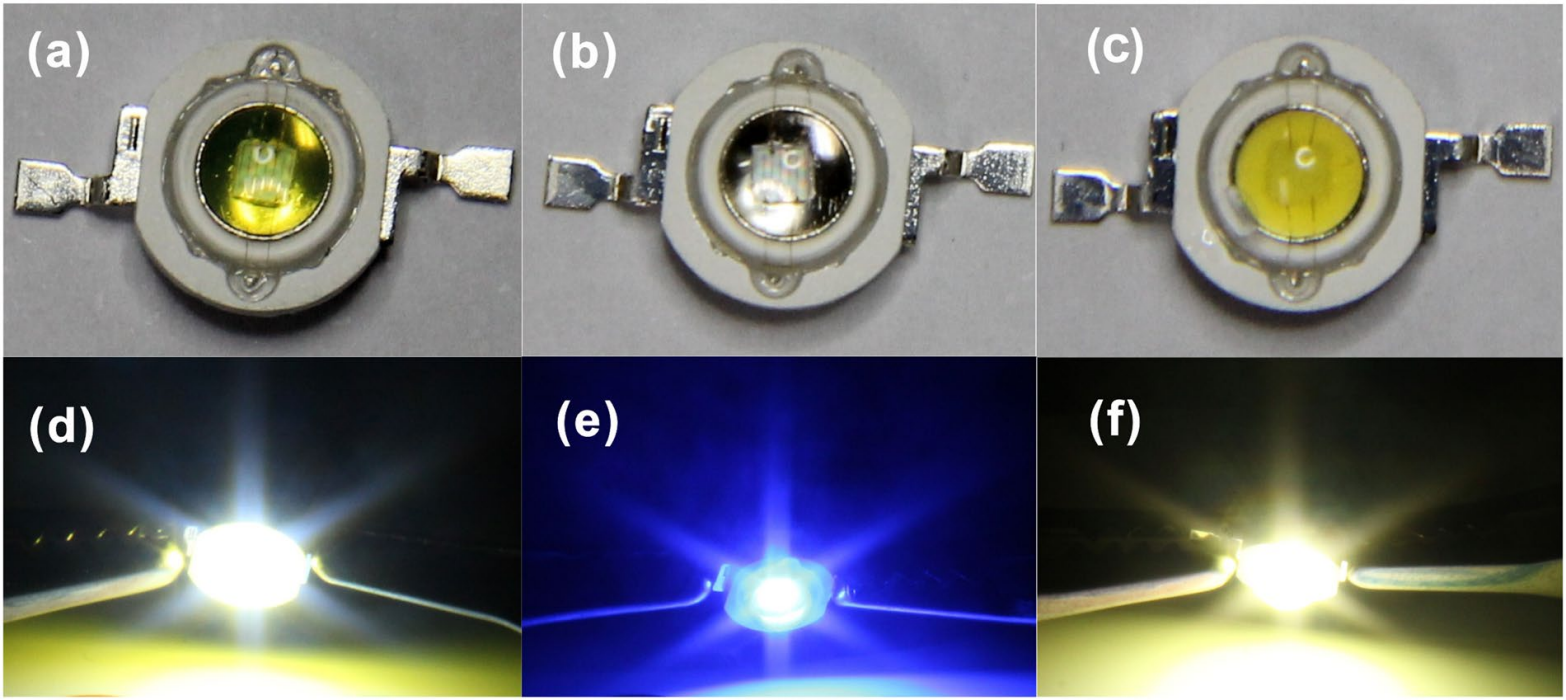
Photographs of LEDs. (**a**–**c**) The top view and (**d**–**f**) EL photographs at 350 mA of (**a**,**d**) SiCDs coated white LED, (**b**,**e**) bare and (**c**,**f**) phosphor white LED based on blue GaN-based LED.

Recently, we have designed and developed a simple method, i.e., one-pot pyrolysis method, for preparing organic pre-functionalized CDs. Especially, silane functionalized CD (SiCD)^35,48^, the first silane pre-functionalized, completely polymerizable nanomaterial and inorganic material, could be bulk self-polymerized or hybrid copolymerized with silanes to form many environmental stable, transparent, and highly luminescent nanohybrid solid Ormosil structures (nanospheres, films, coatings, xerogel glasses, aerogels, and fibers). Stable covalent connection at the molecular level and arbitrary doping (0%–100% scale) of SiCDs in solid nanohybrids could be achieved, and these properties could be easily modulated. These SiCD-based materials prove to be useful in many technologies^35,48–60^, such as bioimaging, laser, solar cell, optical limiting, sensors, photocatalyst, etc. Given that they exhibit surprisingly high PL performance and relatively high QY (55% for SiCD, 88% for SiCD gel glass^35^, and 68% for SiCD-epoxy composite^53^), these materials should be an excellent CCLs. In this study, we fabricated white LEDs (SiCD-WLEDs) using polymerized SiCDs as CCL and encapsulation layer. Compared with the two types of material layers (conventional mixture of a phosphor and polymer matrix), SiCDs were a one-component system. This system could only be drip-coated on bare GaN blue LEDs and subsequently cured to SiCD nanohybrids by thermal polymerization at a modest temperature (<100 °C or room temperature) without adding any curing reagent and catalyst. White, blue, green, yellow, and orange LEDs could be achieved and simply modulated by changing preparation condition as well as doping ratios and amount of coated SiCDs (Figs 1 and 2). It is more important that the SiCD nanohybrids coating exhibit broad color tunability and high CRI for white emission from GaN blue LED. Luminous efficiency was 79.4 lm/W at 350 mA, which was much higher than those of previous best efficacy of organopolymers, QDs or CDs based light-conversion WLEDs, and near that of yellow phosphor based light-conversion WLEDs.

## Results

### Preparation of SiCD based WLED

SiCDs materials were prepared using a modified method reported in literature^35^. SiCDs **1–4** (see Figure S1) were utilized as a light-conversion material for WLEDs excited by commercial blue GaN-based LEDs. A few of drops of SiCDs or SiCDs and organosilane (same as the sample shown in scheme 1) were dripped onto a commercially available blue GaN LED chip (peak emission of ~450 nm) using a transfer liquid gun. SiCDs are tended to hydrolyze and condense to complete bulk polymerization catalyzed by heating and/or trace water in airs^35^. After self- or co-polymerization (with silane) of SiCDs were completed by elevated temperature (80 °C for 2 h, 100 °C for 1 h) or room temperature (6 h) without adding any curing reagent and catalyst, a layer of polymerized SiCDs was formed on LED surface. White light was obtained by mixing the transmitting blue light from the blue LED and yellow-green light emitting from SiCDs excited by a blue LED. Figure 2(a) and (b) show SiCDs and blue GaN based LED device, respectively. From the top view, almost the same visibility of LED chip in SiCD-WLED and blue LED shows excellent light transmittance performance of SiCDs polymerized hybrid coatings. Phosphor WLED device in Fig. 2(c), which LED chip coated with yellow phosphors, has strong light blocking and light leaking for microscale phosphors. The corresponding EL photograph of SiCD-WLED and phosphor WLED at 350 mA is shown in Fig. 2(d) and (f), respectively. True color badge shows good color rendering performance of SiCD-WLED. Figure 2(e) shows the EL photograph of a blue GaN-based LED device at 350 mA with poor color rendering performance. Thus, SiCD-WLEDs could produce warm white light, and SiCDs are superior to those phosphors for high transparency, and absence of light blocking and leakage. As a contrast, a yellow phosphor based light-conversion WLED was prepared by conventional coating and encapsulation process.

### The performance of SiCD-WLEDs

**Figure 3:**
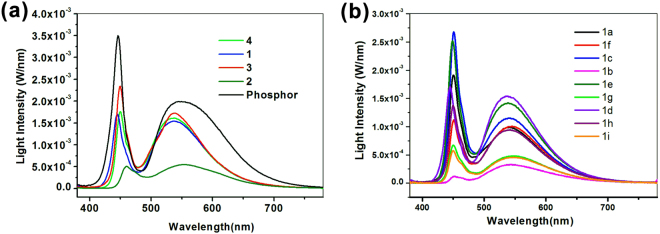
EL spectra of WLEDs. (**a**) Absolute EL spectra of WLEDs based on phosphor and SiCD 1–4 prepared by different silanes; (**b**) Absolute EL spectra of WLEDs based on SiCD **1a–1i** with different preparation conditions (carbon source ratio and pyrolysis reaction time as shown in Table 1).

The corresponding EL spectra of SiCD-WLED are shown in Fig. 3, with emission peaks of blue LED chip and SiCD hybrid coatings were located at 460 and ~550 nm, respectively. The current-voltage (I-V) curves of SiCDs white and blue LED overlapped are shown in Figure S3. SiCDs had no negative effect on the electrical property of LEDs. After coating SiCDs, the intensity of the blue light weakened, accompanied by the presence of a broad band emitting light peaking at ~550 nm (Full width at half maximum = 94–111 nm, FWHM), which mixed with 460 nm LED blue light to obtain white light. The applied voltage and current for LEDs were 3.1 V and 350 mA (working parameters of commercially available GaN LEDs), respectively. By tuning growth parameters of the SiCDs, highly sought white LEDs with Commission International d’Eclairage (CIE) chromaticity coordinates ((0.24, 0.28)-(0.31, 0.43)) for solid-state lighting were obtained. Clearly, with a SiCD layer, CIE of blue LED demonstrated that SiCDs can convert blue light into white light. The best luminous efficiency of SiCD-LED gave 79.4 lm/W at 350 mA, which was much higher than that of the previous best efficacy of organopolymers (23.7 lm/W at 10 mA)^5^, QDs (47 lm/W at 60 mA)^14^ or CDs (42 lm/W at 20 mA)^33^ based light-conversion WLED, and close to that of yellow phosphor based light-conversion WLED (130–230 lm/W at 350 mA)^1^. This finding could be due to silane *in situ* pre-functionalization, covalent connection and dispersion at the chemical molecular level, completely preventable agglomeration and movement of high PL SiCDs^35,61^.

### The control parameters SiCD-WLED

As shown in Fig. 3 and Table 1, absolute EL spectra of encapsulated SiCDs-WLEDs were measured with an integrating sphere and precise rapid spectral radiometer according to the industrial standard. Emission peaks of SiCD **1–4** are located at 541–549 nm. SiCD-WLED and phosphor WLED had color coordinates of (0.3025, 0.4028), (0.3028, 0.4157), (0.3454, 0.4657), and (0.3306, 0.3620) in CIE 1931 color space, respectively. The color temperature (Tc) is measured as 6228, 4455, 6432, 5223, and 5579 K, and the CRI is 71.4, 68.3, 72.6, 61.5, and 70.8. Luminous efficiency is 70.93, 73.39, 79.39, 50.88 and 108.86 lm/W. Thus, broad FWHM (111 nm), luminous efficiency, and light-emitting intensity of SiCD-LEDs were all in the same order of magnitude as phosphor-based WLEDs. All parameters demonstrated that SiCD **3**-based WLED had the best performance. This could be due the ability of SiCD **3** to more faster condense into a gel with greater crosslinking because SiCD **3** contained more methoxysilyl groups than 1, as well as the quicker reaction rate of methoxysilyl groups of SiCD 3 than ethoxylsilyl groups of SiCD **2** and **4** (see Scheme S1).

**Table 1:** The preparing factors and spectral performances of SiCDs excited by 360 nm UV light and the SiCD-WLEDs excited by 456 nm LED at 350 mA current.

CDs	1a	1b	1c	1d	1e	1f	1g	1h	1i	2	3	4	Phoshpor
Amount of citric acid and reaction time	0.5 g 1 min	0.5 g 30 min	0.5 g 60 min	1 g 1 min	1 g 5 min	1 g 30 min	1 g 60 min	1.5 g 1 min	1.5 g 30 min	1 g 5 min	1 g 5 min	1 g 5 min	—
PL peaks (nm) excited by 360 nm UV light	437	439	442	439	440	442	445	442	443	440	443	435	
QY (%)	45	40	32	50	48	39	25	30	28	49	55	43	
Emission peaks (nm) excited by 456 nm LED	542	544	543	541	541	545	549	542	548	553	539	538	550
FWHM (nm)	108	111	104	98	102	103	110	104	111	111	94	97	124
Luminous efficiency (lm/W)	46.07	54.17	15.85	70.93	68.1	49.71	26.06	44.69	22.03	73.39	79.39	50.88	108.86
Tc (K)	8248	4739	7926	6228	9234	6009	5577	6866	5789	4455	6432	5553	5579
Ra	67	60	66.2	58.2	67.1	61.3	64.9	64.3	66.8	59.9	57.2	58.4	70.1
Light conversion efficiency η (%)	27.1	8.3	32.4	39.2	38.5	25.9	13.5	24.9	12.1	16.5	42.8	27.6	72.9

SiCD-LEDs could be achieved and simply modulated by changing the preparation conditions. As the pyrolysis time of SiCD was increased from 1 to 60 min, the color of SiCD and corresponding CD-LED coatings changed, the absorption and emission (PL excited by 360 nm UV light and 459 nm LED as shown in Table 1 and Fig. 3b) shifted to longer wavelengths, QYs and luminous efficiency decreased. From the EL spectra (Fig. 3b and Table 1), the intensity of **1d** white LED was found to be the highest in this series. Luminous efficiency of SiCD **1d** WLED is 70.93 lm/W. Emission peaks of SiCD **1a–1i** were located at 541**–**549 nm. The emitting light of SiCD **1d** had a large light-emitting region from 485 nm to 700 nm and broad FWHM (101 nm). Color coordinates of SiCD-WLED were shown in CIE 1931 color space (Figure S5). SiCD **1d** WLED has a CIE coordinate of (0.2819, 0.3523). Color temperature was measured as 6228 K, corresponding with cool white light, and CRI (Ra) was 69.1. True color badge showed the good CRI of SiCD **1d** WLED.

**Figure 4:**
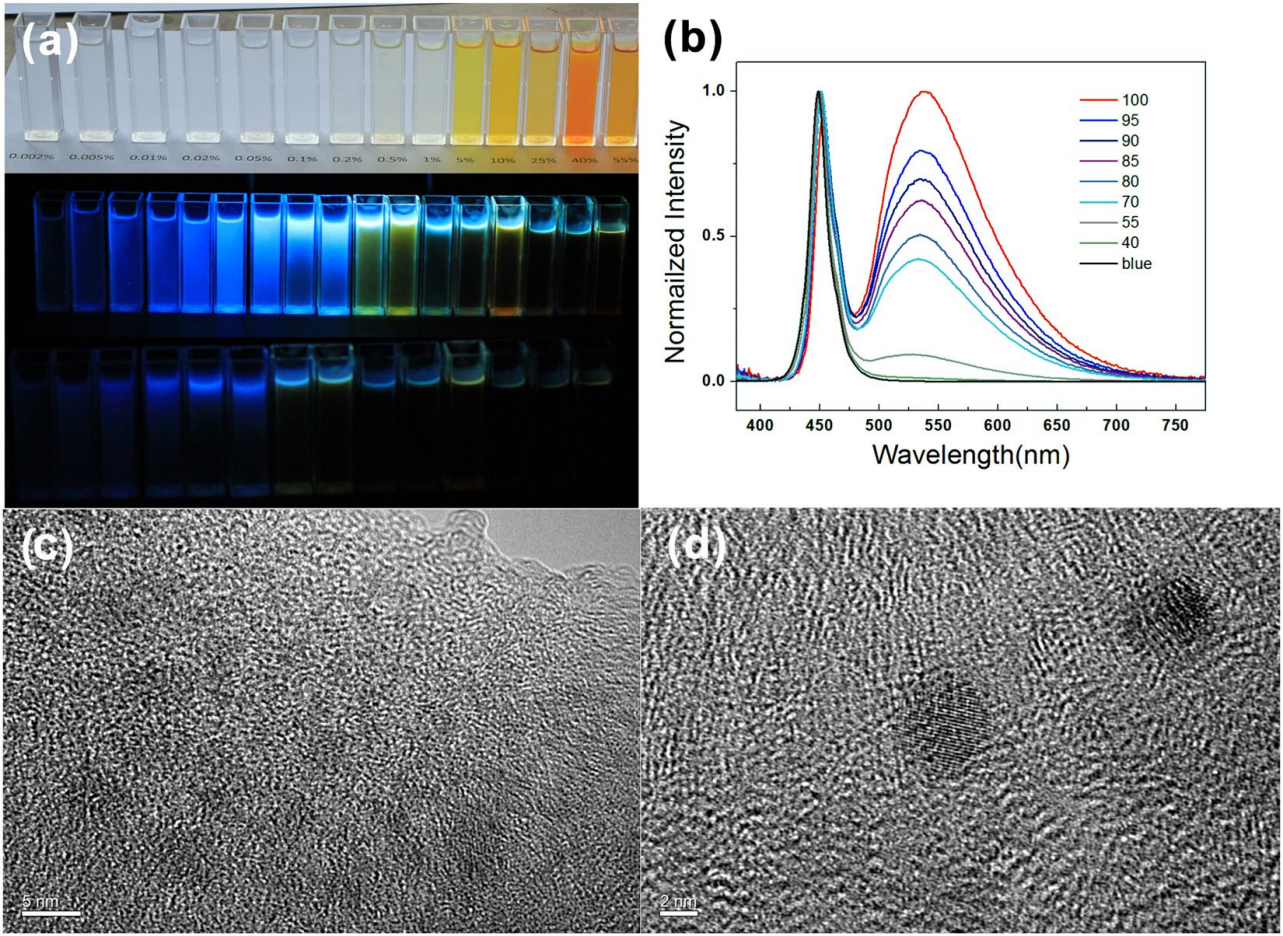
Arbitrarily doped SiCDs. (**a**) Optical photographs of a series of arbitrarily doped (0–100 wt% scale) SiCDs upon visible light (top), 365 nm (middle) UV and 254 nm (bottom) UV light illuminations. (**b**) EL spectra of SiCD white LEDs under various doping volume fractions with methyltriethoxysilane (40–100 wt%). TEM images showing the distribution of SiCDs in coatings. (**c**) close to the edge of the film cross-section on TEM grid, and (**d**) an exemplary location at an inner point across the thickness of cross-section, as is repeated at other locations (scale bar = 5 and 2 nm).

The SiCD emission performance excited by GaN blue LED can be tuned by adjusting the amount of SiCDs as shown in Figure S2. With the increasing of self-polymerized SiCDs coating amount, emission peak intensity relatively increased and emission peak position red-shifted from 535 nm to 539 nm. This red-shift may originate from the self-absorption of SiCDs. Aparts from self-polymerization, SiCDs could polycondense with silanes and silicone. The loading fraction of SiCDs can be easily controlled from 0% to 100% by changing the ratio of SiCDs to silane, and properties of as-obtained SiCD-WLED could be accordingly modulated. As the loading fraction of SiCDs decreased, the following changes were observed. The color of SiCD-based LED under nature light changed from reddish brown to colorless. Luminous efficiency and intensity decreased, and spectra shifted toward longer wavelengths (Fig. 4 and Table 1). SiCD coatings with a high loading fraction (>55 wt %) showed higher luminous efficiency and greater WLED performance.

Figure 4a shows that all SiCDs were amorphous liquid CD fluids and intermiscible with water, common solvents, and silanes. Thus, molecular level and arbitrarily doping (0–100% scale) of SiCDs in most solvent, copolymerized sol-gel Ormosil solid hybrid structures, and blended epoxy resin composites^53^ could be achieved. These properties of prepared solutions and composites could also be accordingly modulated. These solid structures were optically, thermally, and mechanically stable, as well as highly transparent (>80%) in the visible to near-IR region^35^. To study homogeneity and optical quality of produced layers, TEM studies of slides prepared by microtome cross-sectional cutting of SiCD polymerized hybrid coatings were conducted. TEM images taken at different positions of sample demonstrated the uniformity of SiCDs dispersed within host gel (see Fig. 4c,d)^62^. SiCDs were observed be spherical and uniformly distributed in gel without aggregation. As a result, agglomeration and phase separation of SiCDs, which commonly appeared in other most composites, are completely prevented. Resultant hybrids also offered sustained and even surpassed PL performance. SiCDs can be homogeneously dispersed in gel layer to avoid unnecessary optical absorption losses. Therefore, SiCDs had well compatibility (arbitrarily doping) with silicone and epoxy matrix typically used to fabricate CCLs in the LED industry.

**Figure 5:**
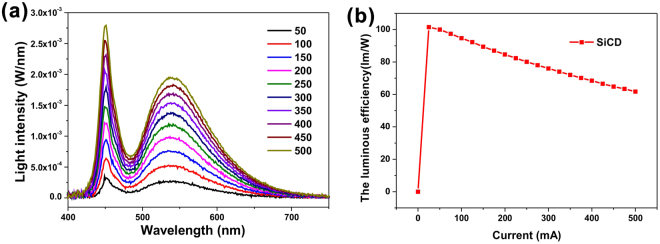
The optical performance of WLED. (**a**) Absolute EL spectra and (**b**) luminous efficiency of SiCD **3** based WLED under various forward currents (50–500 mA).

EL spectra of SiCD-WLEDs under various forward currents are shown in Fig. 5a. Emission peaks of blue LED chip and SiCDs were located at 450 nm and 538 nm at 350 mA, respectively. Both blue and green-yellow emission intensities steadily increased with increased current, revealing that LEDs had stable light-conversion and color quality. The luminous efficiency of SiCD-WLEDs at different forward currents is shown in Fig. 5b. The luminous efficiency of SiCD WLED was 100.04 lm/W under 50 mA, whereas that of the blue LED was 20.05 lm/W. Although a slight decrease in luminous efficiency from 100.04 to 79.39 lm/W was observed when forward current increases from 50 mA to 350 mA, luminous efficiency of SiCD-WLED was higher than those of reported organopolymer^5^, QD^14^, and CD^33^-based LEDs. The light conversion (blue-to-SiCD emission) efficiency of SiCD white LED slowly decreased from 44.7% to 39.5% with increased current. The light conversion (blue-to-phosphor emission) efficiency of phosphor white LED were maintained from 72.6% to 72.5% with increased current. Results revealed that SiCD exhibited good light conversion stability compared with the recently reported QD-based WLED.

The CIE coordinates of LED (Figure S5) was changed from (0.2960, 0.4086) to (0.2949, 0.3879), and no obvious change of CIE coordinates was observed at different applied currents, indicating greater color stability of output light. Blue LEDs with the same parameters except wavelength were also used as exciting light sources. A 459.1 nm LED and a 445.1 nm LED were coated with the same amount of SiCDs. As shown in Figure S5, Tables S1 and S2, the peak wavelength of emission light was maintained at 539 nm. In short, within excited wavelength and various forward current ranges, energy conversion efficiency and peak wavelength of emission light were maintained.

Most importantly, after CD incorporation, most blue emission was absorbed by SiCDs and down-converted to green and yellow lights, thereby creating white-light emission from specific GaN based LEDs. If we assume that CD incorporation did not affect extraction efficiency from GaN structure, by integrating the spectra of a GaN LED and a SiCD-WLED in excitation (blue) and emission (green, red) areas, down-conversion quantum efficiency in SiCD-WLED would be 42.8%. This value very well agreed with our CDs’ QY. Figure S4 compares photographic images of blue and white emission from two different locations (region A and B after CDs incorporation) along the device. By analyzing emission spectrum, the CIE chromaticity coordinates for SiCD-WLED emission yielded values of x = 0.33 and y = 0.21. Color temperature was 5030 K, and CRI (Ra) was 74. Notably, the proximity of CDs to LED active blue-emitting region led to rather efficient optical pumping because of high “on-site” pumping intensity and increased optical path length of pumping photons in a diffusive nanoporous structure, as well as the superposition of all of RGB-emitting sources. This phenomenon should be contrasted to conventional solid-state lighting luminaire structures, wherein phosphors were typically placed in a separate space to the LED wafer, and were far away from the pump source, such as the inner surface of encapsulation cap.

## Discussion

In summary, green and low-cost polymerizable SiCDs prepared by one-pot pre-functionalized method were used as the color conversion and encapsulation layer of commercially available GaN blue LED. In contrast to the conventional mixture of a phosphor and polymer matrix, SiCDs were individually polymerized one-component system, which was drip-coated and bulk polymerized on GaN LEDs. The SiCDs exhibit excellent light converting properties as compared to organopolymers, semiconductor-based QDs or other CDs, and are close to that of yellow phosphor based light-conversion WLED. The organic–inorganic hybrid siloxane component and covalently bridged structure of SiCDs hybrid coatings induced environmental stability, broad band PL, high transparency (no light blocking and light leakage), and arbitrary doping. Importantly, the performance of SiCD-LED could be controlled by coating amount, arbitrary doping concentration of SiCDs hybrid coatings, preparation conditions, surface functional groups, and types of SiCDs. Energy conversion efficiency and emission wavelength of SiCD-based LED were not insensitive to excitation wavelength and working current of blue GaN-based LEDs. These results demonstrate that a CD CCL with an appropriate structure will hope to replace phosphors color conversion and encapsulation layers simultaneously for LED applications. The unique and excellent solubility, flexible chemical and mechanical processability, nontoxic nature, arbitrary polymerization doping, easy surface- functionalization and hybridization^63–67^, as well as white-light emission from SiCDs will make this material promising for a wide range of optoelectronic devices.

## Materials and Methods

### Materials

The silanes were purchased from Aldrich and Beijing Shenda Fine Chemical Co. Ltd. Commercial YAG Phosphor (0911008) was purchased from Xiamen kemingda science and Technology Co., Ltd. The other reagents were obtained from the Chinese Reagent Corporation and were of analysis grade. All the reagents were used as received without further purification. The Blue LEDs with a size of 45 mil*45 mil and patterned sapphire substrate emitted blue light of about 459 nm were used as the exciting light sources. The LED chips were manufactured and supported by Shandong Inspur HuaGuang Optoelectronics CO., LTD.

### Preparation of the SiCDs

10 mL organosilane (Figure S1) was placed into a 100 mL three-necked flask, and degassed with nitrogen for 15 minutes. Upon reaching the appropriate temperature, appropriate weight (Table 1) citric acid was quickly added to the solution under vigorous stirring, and then kept for 1–60 min under the temperature. The final products SiCD **1–4** were purified by precipitating with petroleum ether three times or using silica gel column chromatography.

### Preparation of the SiCD-LEDs

About 5 µL SiCDs was dropped onto a blue LED chip fixed on a LED basal plate with a 20 µL pipette filler or transfer liquid gun. LEDs coated with SiCDs or/and methyltriethoxysilane were heated at 80 °C for 2 h in an oven or at room temperature for 6 h to solidify SiCDs. The LEDs were covered with semicircle PMMA caps. The gap between the LED and cap was filled with silicone resin. Then, LEDs were heated at 100 °C for 1 h to solidify the silicone resin. Phosphor based white LED was manufactured based on the same process.

### Characterization

Fluorescence spectra were recorded using F-4500 fluorescence spectrophotometer. UV-Vis spectra were measured on UV-1601PC UV-visible spectrophotometer. Samples for scanning electron microscopy (SEM) were coated with gold and the energy dispersive X-ray spectroscopy were measured using Hitachi S-4300 field emission scanning electron microscope. Transmission electron microscopy (TEM) images were obtained from JEM 2100 F (Japan, JEOL) operating at 200 kV accelerating voltage. The absolute EL spectra of encapsulated SiCD-WLEDs were measured by a precise rapid spectral radiometer (EVERFINE HAAS-2000) with an integrating sphere according to the industrial standard. The LEDs were driven at rated current of 350 mA. All optical and electrical parameters were automatically calculated by the radiometer based on the absolute EL spectra.

## Electronic supplementary material

Supporting Information
