# Supplementary material for: White Light-Emitting Diodes Based on Individual Polymerized Carbon Nanodots
Source: Sci Rep. 2017 Sep 22;7:12146. doi: 10.1038/s41598-017-12083-2 (PMC5610343; doi:10.1038/s41598-017-12083-2)
Supplement: Supplementary file 1 — Supporting Information [file 41598_2017_12083_MOESM1_ESM.pdf]

Supporting Information

White Light-Emitting Diodes Based on Individual Polymerized Carbon Nanodots

Concise running title: White Light-Emitting Diodes Based on Carbon Nanodots

Zheng Xie[1,†], Zhengmao Yin[2,3,†], Yongzhong Wu[2], Chunyan Liu[1,*], Xiaopeng Hao[2,*], Qingqing Du[1,2] and Xiangang Xu[2,4]

[1]Laboratory of Photochemical Conversion and Optoelectronic Materials, Technical Institute of Physics and Chemistry, Chinese Academy of Sciences, No.29 Zhongguancun East Road, Haidian District, Beijing 100190 (P. R. China).

[2]State Key Laboratory of Crystal Materials, Shandong University, 27 Shandanan Road, Jinan, 250100 (P. R. China).

[3]College of Materials Science and Engineering, Qingdao University of Science and Technology, Qingdao 266042, (P. R. China).

[4]Shandong Inspur HuaGuang Optoelectronics CO., LTD, 1835 Tianchen Street, High-tech Zone, Jinan, 250101 (P. R. China).

*Address correspondence to Xiaopeng Hao (xphao@sdu.edu.cn) and Chunyan Liu (cyliu@mail.ipc.ac.cn)

†These authors (Zheng Xie and Zhengmao Yin) contributed equally to this work.

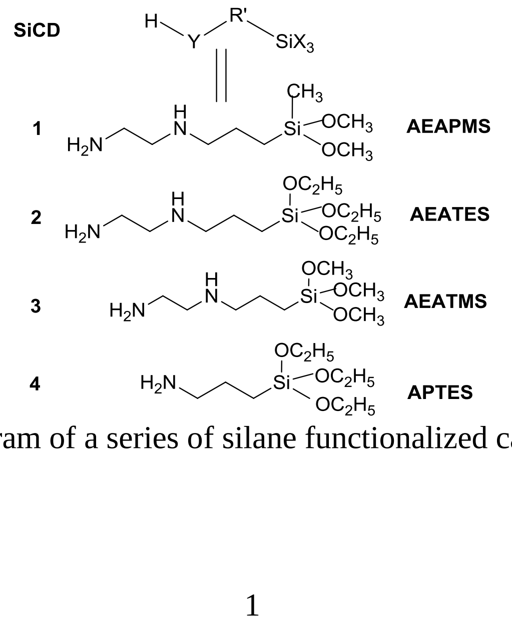

Figure S1. Schematic diagram of a series of silane functionalized carbon dots

Table S1. Spectra data of CDLED at 350 mA current and excited by 456 nm LED

CD	**1e**	phosphor
Emission		
Peaknm		
luminous efficiency lm/W	69.82	95.68
TcK	5727	5245
Ra	61.2	76.7
η (%)	39.0	69.5

Table S2. Spectra data of CDLED at 350 mA current and excited by 444 nm LED

CD	**1e**	phosphor
Emission		
Peaknm		
Luminous efficiency lm/W	70.5	94.75
TcK	5455	5861
Ra	61.5	67.5
η (%)	39.1	64.2

η (%): Energy conversion efficiency of SiCDs excited by blue LEDs.

The calculation of light conversion efficiency (η) of SiCDs excited by blue LEDs:

To ignore the influence of the same encapsulation, the energy conversion efficiency of SiCDs excited by blue LEDs was calculated as follows.

Ⅰ. Exciting energy of blue LED = total energy of blue LED plus energy of blue light in the white light. spectral integral area of blue LED plus integral area of blue light of SiCD white LEDs.

Ⅱ. Emission light energy = integral area of the spectrum except blue light of SiCD white LEDs.

Ⅲ. Energy conversion efficiency = emission light energydivided by exciting energy.

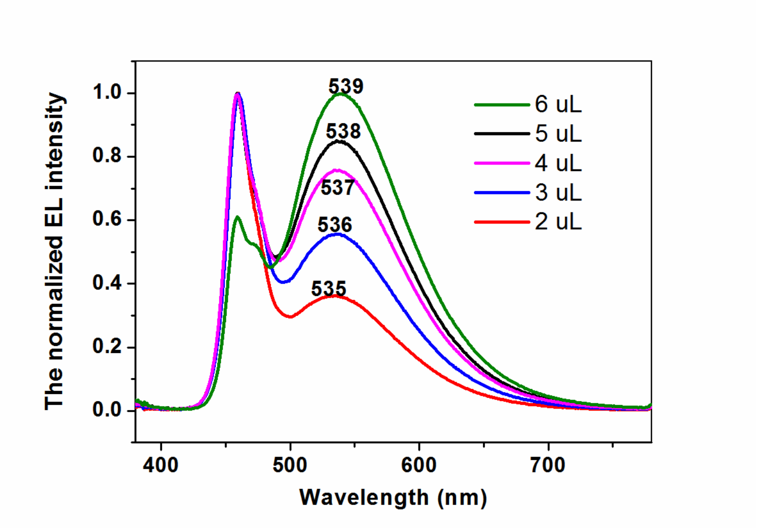

Figure S2. The normalized EL spectra of white LEDs vs SiCDs coating amount.

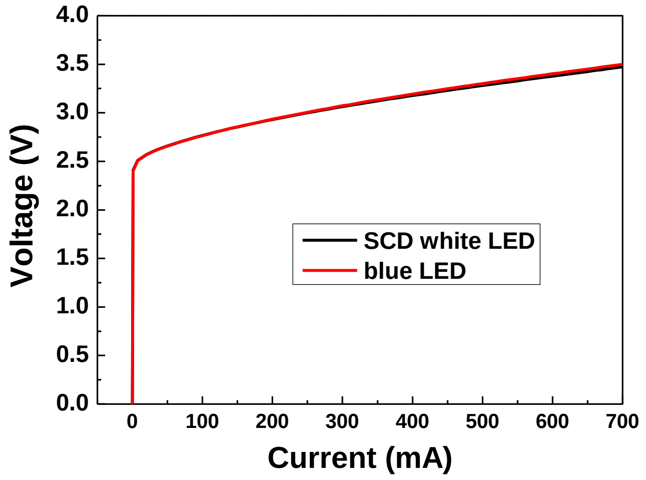

Figure S3. I-V curves of blue LED and SiCD white LED.

The current-voltage (I–V) curves of SiCD white LED and blue LED overlapped. SiCD have no negative effect on the electrical property of LEDs.

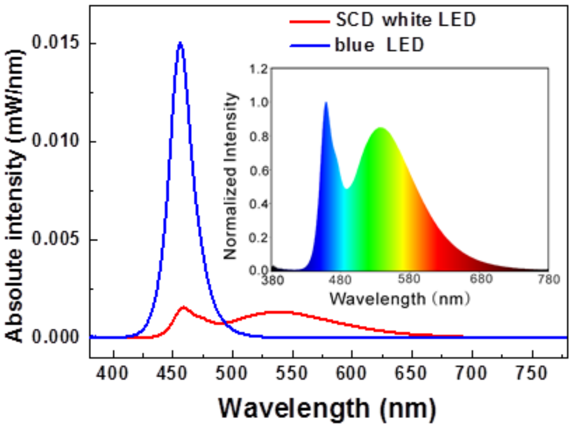

Figure S4. EL spectra of SiCD white LED and blue GaN-based LED under 350 mA. The insert shows the normalized EL spectrum of SiCD white LED at 350 mA.

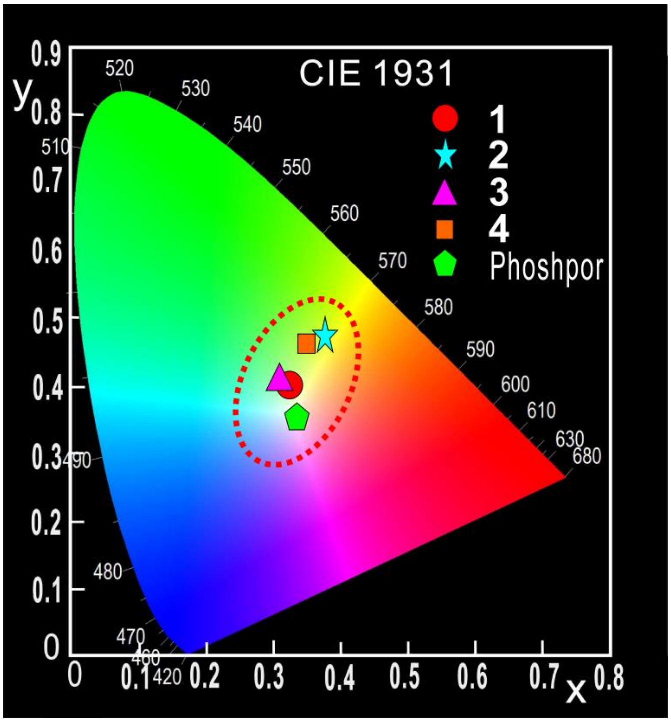

Figure S5. CIE color coordinates of SiCD-WLEDs excited by blue LEDs and blue LED at 350mA. The dashed circle at the center of CIE represents the white emission region of SiCD-WLEDs and phosphor based WLEDs.
